# SpecNet: A Spatial Network Algorithm that Generates a Wide Range of Specific Structures

**DOI:** 10.1371/journal.pone.0042679

**Published:** 2012-08-02

**Authors:** Jenny Lennartsson, Nina Håkansson, Uno Wennergren, Annie Jonsson

**Affiliations:** 1 Systems Biology Research Centre, University of Skövde, Skövde, Sweden; 2 Department of Physics and Measurement Technology, Biology and Chemistry, Theory and Modelling, Linköping University, Linköping, Sweden; Universidad de Zarazoga, Spain

## Abstract

Network measures are used to predict the behavior of different systems. To be able to investigate how various structures behave and interact we need a wide range of theoretical networks to explore. Both spatial and non-spatial methods exist for generating networks but they are limited in the ability of producing wide range of network structures. We extend an earlier version of a spatial spectral network algorithm to generate a large variety of networks across almost all the theoretical spectra of the following network measures: average clustering coefficient, degree assortativity, fragmentation index, and mean degree. We compare this extended spatial spectral network-generating algorithm with a non-spatial algorithm regarding their ability to create networks with different structures and network measures. The spatial spectral network-generating algorithm can generate networks over a much broader scale than the non-spatial and other known network algorithms. To exemplify the ability to regenerate real networks, we regenerate networks with structures similar to two real Swedish swine transport networks. Results show that the spatial algorithm is an appropriate model with correlation coefficients at 0.99. This novel algorithm can even create negative assortativity and managed to achieve assortativity values that spans over almost the entire theoretical range.

## Introduction

Network modeling is frequently used in different areas of study, such as biology, economics, epidemiology, and social science. Both empirical and theoretical networks are investigated, and questions that can be of interest in theoretical explorations of network structures can, for example, consider how the structure of a farm network affects the transmission of some disease or how the connections in an ecological food web influence the dynamics of the species involved. To clarify, when we refer to networks, we mean networks that may contain separated components or even isolated nodes. Isolated nodes are nodes without any contacts to other nodes. In real-world dynamical networks, like for example networks of animal transports, it is possible that isolated nodes exist within a specific time window and therefore such nodes were allowed in this study. To address network issues, it is important to be able to generate a broad range of different structures that can capture all possible structures of empirical networks. Accordingly, it is essential to have a network-generating algorithm that can produce a wide array of desired structures.

Network generating algorithms have been developed and investigated by for example Asano [1], Watts and Strogatz [2], Barabási and Albert [3], Eubank et al [4], Keeling [5], Christley et al [6], Shirley and Rushton [7], Bansal et al [8], Boily et al [9], Badham and Stocker [10], and Håkansson et al [11]. Indeed, a fairly large number of algorithms have been introduced for the indicated purpose, but it seems that discussion is needed regarding the ranges of network structures that the algorithms are able to create. For example, Badham and Stocker [10] produced networks with assortativity of 0.0 to 0.3 and clustering coefficient between 0.0 and 0.4, which represent small values of the theoretical ranges (between 0.0 and 1.0) of these measures. In another study, Badham and colleagues [12] analyzed the ability of Keeling's spatial algorithm [5], [13] to generate different structures of social networks and observed a broad spectrum of structures, such as clustering between 0.09 and 0.85, and assortativity between 0 and 0.9. The upper limits for the average clustering coefficient and positive assortativity obtained using Keeling's algorithm agrees with, and is even slightly higher than, the values generated in this study, but Keeling's algorithm has limitations regarding negative assortativity [11]. The studies performed by Badham et al [10], [12] using this algorithm did not focus on networks with negative assortativity, probably because they focused on social networks, which most often have positive assortativity.

Distance is an important aspect in many networks. For instance, in transportation networks, distance can affect costs and the time it takes for a vehicle to travel between nodes, which can influence the probability that a particular transport will occur. Other examples where distance plays a significant role include internet networks [14], [15], mobile phone networks [16], [17], disease transmission networks, [5], [7], [10], [18], [19], social networks [20], [21], and neural networks [22]. In non-spatial networks the spatial location of the nodes is unimportant, since the nodes exist only in an abstract space, such as in citation networks or biochemical networks [23]. Here, we present an algorithm that makes it possible to obtain networks in which there is a multitude of structures, where nodes are located in space, and link forming depends on a probability distribution based on Euclidian distances between nodes. We refer to networks formed in this way as spatial networks. The algorithm we apply is called SpecNet, and it can generate networks with a specified number of nodes and mean degree that can be tuned to the desired assortativity, clustering, and/or fragmentation. This is possible by adjusting just a few input parameters.

We analyze the performance of the SpecNet algorithm to generate wide ranges of network structures, and also compare it with an extended version of the non-spatial configuration model (CM) algorithm [24], [25], [26], [27], [28], [29], [30], which we designate CMext.

The CMext algorithm was chosen as comparison to the SpecNet algorithm because of its ability to generate networks with some desired structures; degree-dependent clustering and assortativity. Even though the CMext algorithm is based on a non-spatial method, we apply it to generate networks with specific structures originating from spatial networks. We use the algorithm to generate random networks with a specified level of these structures given an *a priori* degree distribution. More specifically, we analyze the capability of the SpecNet and CMext algorithms to generate networks with desired values of clustering coefficient, degree assortativity, fragmentation index, and mean degree. We examine the ranges of these network structures that the two algorithms are able to produce and what combinations of values can be obtained.

The network structures we have chosen to compare and discuss have previously been shown to be important for predictions of disease transmission. In disease transmission networks, the node degree has a considerable impact on the risk that an individual node will be infected and also influences the risk of spread of the infection [18], [31]. Ames et al [32] showed that, for very sparse (networks with low mean degree) or very dense networks (networks with high mean degree), it is sufficient to have knowledge of the degree distribution in order to make predictions about disease transmission. However, for networks with intermediate link density (networks with mean degree of 5–11), it is also necessary to have information about other network structures, such as clustering and mean path length (see also [33]. It has been concluded that clustering and degree assortativity plays a decisive role in the risk of epidemics. Badham and Stocker [10] have shown that the final size of epidemics on networks decrease with increasing values of clustering coefficient or of degree assortativity and Keeling [5], [13] concluded that epidemics are less likely in networks with high clustering. Barthélemy [34] has argued that the level of clustering and degree assortativity must be included in a complete characterization of networks, with an example of airline network structures.

In this article, we discuss the usefulness of the SpecNet algorithm for generating new networks with specific structures or for reproducing available empirical networks. We also compare our spatial SpecNet algorithm with the non-spatial CMext algorithm. As an example, we test the possibility of applying these two algorithms to regenerate two different empirical networks for swine transports in Sweden, one for transports between farms and one for transports to slaughterhouses.

## Materials and Methods

### Network Measures

Networks can be described and categorized using different measures [35]. To test the performance of the SpecNet and CMext algorithms, for each generated network we calculated four network measures: clustering coefficient [2], degree assortativity [36], fragmentation index [37], [38], and mean degree [21], [35]. Calculations were done in MATLAB (version 7.4) and Python (version 2.6.5).

The clustering coefficient is the number of links that connect the neighbors of a node to each other divided by all possible connections between the neighbors. The theoretical range of the measure is from 0 to 1, where 1 indicates that most of the neighbors of a node are likely to be connected to each other. Here, the average clustering coefficient for the whole network was calculated.

Degree assortativity is a measure of whether nodes with similar degrees are connected to each other (a value close to 1), or if nodes with different degrees are connected to each other (a value close to −1). An assortativity value of zero indicates that connections between nodes are independent of node degree.

The fragmentation index measures the extent to which the network is disconnected, through measuring the proportion of unreachable node pairs. The measure also considers the sizes of the disconnected components. Fragmentation index ranges on a scale of 0 to 1, where 0 means that the network is connected, and a higher value indicates that the network is fragmented. A value of 1 corresponds to a completely fragmented network without links.

Degree is the number of links that are connected to a node, and the mean degree is the mean for all nodes in the network. The mean degree for a network generated with the SpecNet algorithm is indirectly given from the start, because it can be calculated from the link density (*l_d_*), which is also set from the start. Link density, *l_d_*, represents the actual connections, *L*, in a network as a proportion of all theoretical possible links in that network ([21], eq.1).


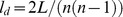
(1)

Here, *n* represents the number of holdings in the network. For networks generated by the CMext algorithm, the mean degree can be calculated from the degree distribution.

### Network generation algorithms

SpecNet represents further development of the algorithm described by Håkansson et al [11], which was capable of generating the desired degree, clustering, and fragmentation, but failed to produce the desired wide range of degree assortativity, especially disassortative networks. The CMext algorithm was first presented by Serrano and Boguñá [29] and further developed by Weber and Porto [30] and Pusch et al [28]. CMext follows the same principle as the CM algorithm [24], [25], [26], [27], except that, in addition to degree distribution, it includes specification of degree-dependent assortativity and clustering. For practical reasons (i.e. simplicity of managing large amount of input and output data and making result figures), both SpecNet and CMext have, in this study, been implemented in MATLAB (version 7.4) and run by us. The tested algorithms generated networks with undirected links; self-loops were not allowed in the runs, and a node pair could be connected by only one link.

### The spectral network algorithm SpecNet

The algorithm described by Håkansson et al [11] uses spectral methods to arrange nodes in a spatial landscape, i.e. generating a node landscape. Links are formed using a probability distribution function, *Prob(i,j)*, and the probability of having a link between, say, node *i* and node *j*, *Prob(i,j)*, depends on the Euclidean distance, *d_ij_*, between the nodes. Here, we have further developed SpecNet to generate a broader spectrum of network characteristics, particularly negative assortativity (disassortative networks) since the previous version of this algorithm [11] did not manage to generate assortativity values below −0.1. Thus, a given proportion of the nodes were assigned as focal nodes (*F*), which means that the nodes were divided into two different classes: regular nodes and focal nodes. We also added a focal scale factor (*Fsf*), which is a parameter that regulates the probability of connections between regular nodes and focal nodes. To achieve negative assortativity, the probability of connection between two nodes, say *i* and *j*, *Prob(i,j)* is greater in the case that *i* is a regular node and *j* is a focal node (or vice versa) than if both *i* and *j* are focal or regular nodes. This implies that all focal nodes have the same underlying probability for connection, for a given distance *d_ij_*. Examples of focal nodes are farms that trade large numbers of animals in a transport network or persons that have many contacts in a social network. Here, the focal nodes were chosen randomly among the nodes. The introduction of the focal nodes results in that SpecNet has some similarities to Keelings algorithm [5], in sense that both algorithms dived the nodes into two classes. Below, we present the two main steps in the SpecNet algorithm: (*1*) spatial node distribution and (*2*) link formation.

### Spatial node distribution

The spatial node distribution is created by using FFT (fast Fourier transformation) to scale a random matrix (*L_random_*) that involves spectral methods (equations 2–5). Note that equation 4 should contain X, not the magnitude of X, which was a misprint in the article published by Håkansson and colleagues [11]. At first, a matrix, *L_random_*, with random values from a Gaussian distribution with mean of 0.5 and a standard deviation of 2, *N*(0.5, 2), is generated (equation 2). The matrix size (*L_size_*) of *L_random_* is chosen according to Håkansson et al [11], and here we used a size of 100×100. The *L_random_* matrix is transformed to the frequency domain using fast Fourier transformation (equation 3). The amplitudes of the function in the frequency domain are scaled (equation 4). The scaled matrix (*L*_scaled_) is a two-dimensional 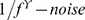. The continuity parameter gamma (*γ*) determines the spatial degree of aggregation between the nodes, where a value of zero results in a random pattern and a higher value, of for example two, ends up in an aggregated node landscape. The *L*_scaled_ matrix is digitalized to a node landscape by giving a chosen number of nodes (*n*) the same coordinates as the index of the elements with highest values of *L_scaled_*.


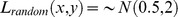
(2)


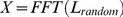
(3)


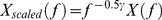
(4)


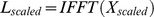
(5)

### Link formation

Links are added to the network one by one until the desired link density, *l_d_*, is achieved. *Prob(i,j)* (equation 6) describes the probability of having a link between two nodes, *i* and *j*, at Euclidean distance *d_ij_*. To avoid edge effects periodic boundaries are used, which means that the left edge of the network is considered connected to the right edge and the upper edge connected to the lower edge. The K in equation 6 is a constant that is recalculated after every draw of a link to keep the total probability sum equal to one. The probabilities of links already drawn are set to zero. The parameters kurtosis (κ) and standard deviation (*σ*) of the probability kernel are functions of parameters *a* and *b* (equations 7 and 8). Kurtosis determines the shape of the probability distribution and the standard deviation (*σ*) controls the variance of the kernel. We use a value of kurtosis corresponding to the exponential distribution, which means that there is substantial probability of links at short distances between nodes. Probabilities of links between regular and focal nodes are increased by the *Fsf*; this is done in equation 6. To investigate the impact of the proportion of focal nodes (*F*) in the network, we vary this amount (see Table 1 for values of all the parameters used). The function Γ, used in equation 7 and 8, is the gamma distribution.


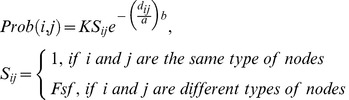
(6)


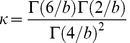
(7)


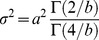
(8)

**Table 1:** Specific parameters and values used in the SpecNet algorithm.

*l_d_*	*γ*	*σ*	*Fsf*	*F*
0.01	0.0	0.01D	10	0.00
0.05	0.5	0.10D	100	0.05
0.10	1.0	0.20D	1000	0.10
	1.5	0.30D		0.15
	2.0			0.20

Parameter *l_d_* is the proportion of links in the networks, *γ* controls the degree of aggregation between nodes, *σ* is the standard deviation of dispersal kernel where D = 100√2 is the diagonal of the landscape, *Fsf* regulates the probability of connections between regular nodes and focal nodes, and *F* is the amount of focal nodes in the networks. Other parameter values used in the network-generating process were as follows: number of nodes (*N*) = 500, matrix size (*L_size_*) = 100×100, and kurtosis (κ) = 10/3.

### The CMext algorithm

The CMext algorithm was implemented in MATLAB according to the scheme published by Pusch et al [28]. As input, the algorithm needs the following: the degree distribution (including number of nodes), the number of connections between nodes with degrees *j* and *k* (i.e., degree dependent assortativity), and information about triangle edges constituted by nodes with degree *k* (i.e., degree dependent clustering). Networks are generated by building triangles of links between three nodes, one at a time, according to the input data. For comparison of the algorithms, we used degree distribution from the networks generated in SpecNet as input data. CMext tries to generate networks with specified degree distribution and clustering, but, after a set number of trials (here 1000), links are randomly distributed between nodes.

### Performance Test of Alogorithms

The performance of the SpecNet algorithm was tested by generating networks with different combinations of the input parameters (Table 1). We kept the number of nodes (*n*) and kurtosis (κ) constant, because our previous study [11] had indicated that these two parameters have little impact on the range of network structures that the SpecNet algorithm can generate. The ranges of the other input parameters used were: *l_d_* (0.01–0.1), *γ* (0–2), *σ* (0.01–0.3), *Fsf* (10–1000), and *F* (0–0.2). We tested a total of 900 combinations of parameter values with 50 replicates for each combination, rendering 45000 networks in total. Data on degree distributions in these networks were then used as input for performance test of the CMext algorithm.

We performed a 5-way ANOVA (ANalysis Of VAriance) and compared the mean sum of squares (MS) to determine the extent to which obtaining desired networks was affected by the parameters: *l_d_*, *γ*, *σ*, *Fsf*, and *F*. The ANOVA was performed in MATLAB (version 7.4) using the function “anovan” with model type “interaction”.

### Empirical Networks

To test and illustrate how empirical networks can be reproduced by the algorithms, we considered two Swedish swine transport networks including a total of 2539 nodes (farms) each. The measured structures we aimed to regenerate were degree assortativity, mean clustering coefficient, and fragmentation index. The two empirical networks were based on data from 2008 provided by the Swedish Board of Agriculture. One network was for transports between farms and consisted of 8019 links (movements), and the other was for slaughterhouse transports and comprised 3035 links. Both networks had negative assortativity, and, as expected, the slaughterhouse network was more disassortative than the network of animal transports between farms. Both networks showed a low level of clustering. The network of transports between farms had a higher fragmentation index (i.e., was more fragmented) than the slaughterhouse network.

As input in the SpecNet algorithm, number of nodes (*n*), link density (*l_d_*) and gamma (*γ*) were already given by calculations from the empirical datasets. To determine appropriate values for the additional input parameters we used the specific empirical network characteristics given in Tables 2 and 3 together with the figures and tables outlining the results of the performance test in this study. As input in the CMext algorithm we used the degree distribution of the empirical networks. Inasmuch as both algorithms include stochasticity, the regeneration was repeated 200 times for each parameter combination in SpecNet and 200 times for each empirical network in CMext. With the SpecNet algorithm, we investigated combinations of parameters and choose to present the result as the parameter combination with the smallest sum of errors, which was found by searching for the minimum absolute difference between the generated values and the empirical values for the calculated network measures.

**Table 2:** Statistics of generated networks.

Network measure	Algorithm	*γ*	min	1% quartile	10% quartile	median	90% quartile	99% quartile	max
Clustering coefficient	SpecNet	[0–2]	0.00	0.00	0.01	0.14	0.61	0.76	0.76
	SpecNet	0	0.00	0.00	0.01	0.13	0.58	0.58	0.58
	Previous	[0–2]	0.01	0.01	0.02	0.14	0.59	0.73	0.77
Degree assortativity	SpecNet	[0–2]	−0.98	−0.97	−0.90	−0.36	0.60	0.73	0.75
	SpecNet	0	−0.98	−0.98	−0.94	−0.42	0.45	0.53	0.53
	Previous	[0–2]	−0.02	−0.01	0.01	0.23	0.68	0.77	0.79
Fragmentation index	SpecNet	[0–2]	0.00	0.00	0.00	0.00	0.16	0.89	0.92
	SpecNet	0	0.00	0.00	0.00	0.00	0.08	0.11	0.12
	Previous	[0–2]	0.00	0.00	0.00	0.00	0.04	0.86	0.94

Statistics of networks generated by the SpecNet algorithm and the Spectral network algorithm with different values of the landscape parameter gamma. With “Previous” we refer to the Spectral network algorithm in Håkansson et al [11].

**Table 3:** Measures of the network of between-farm transports of swine in Sweden 2008.

		Clustering coefficient	Degree assortativity	Fragmentation index
Empirical network		0.04	−0.15	0.47
SpecNet	Generated	0.06	−0.16	0.41
	Min	0.05	−0.44	0.10
	Max	0.14	−0.01	0.77
	Std	0.02	0.07	0.13
			0.07	0.13
CMext	Generated	0.00	−0.03	0.00
	Min	0.00	−0.03	0.00
	Max	0.00	0.02	0.09
	Std	0.00	0.01	0.00

Empirical values as well as values generated by the SpecNet and CMext algorithms are included. The statistics on generated values from the algorithms are calculated on average values of 200 replicates of each tested combination of parameters for respectively algorithm. Std = standard deviation.

## Results

**Figure 1:**
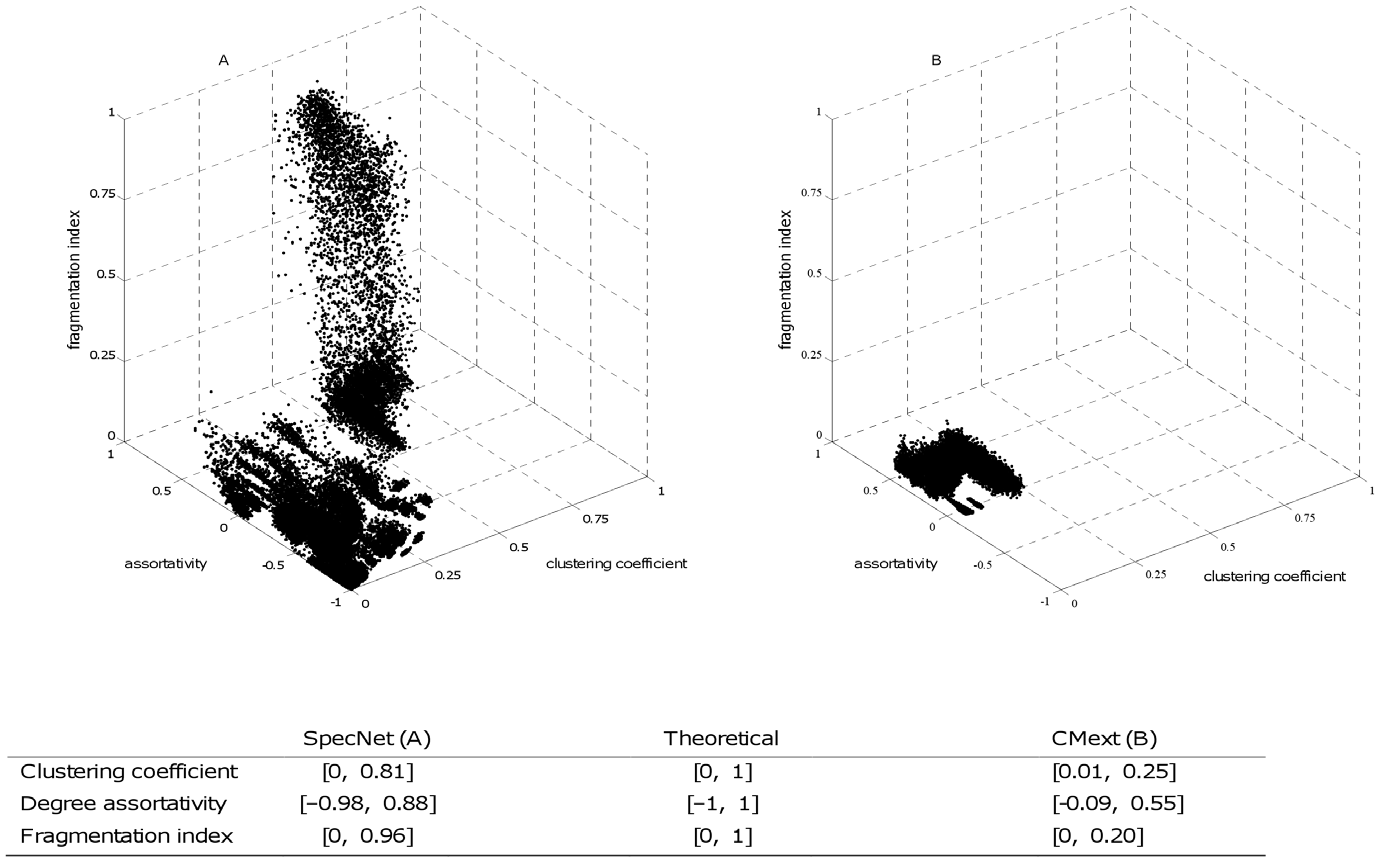
Degree assortativity, clustering coefficient, and fragmentation index. Degree assortativity, clustering coefficient, and fragmentation index values generated by the SpecNet algorithm (A) and the CMext algorithm (B). The theoretical ranges of the network measures, as well as the ranges of values of the measures generated with the SpecNet algorithm and the CMext algorithm are shown in the table.

**Figure 2:**
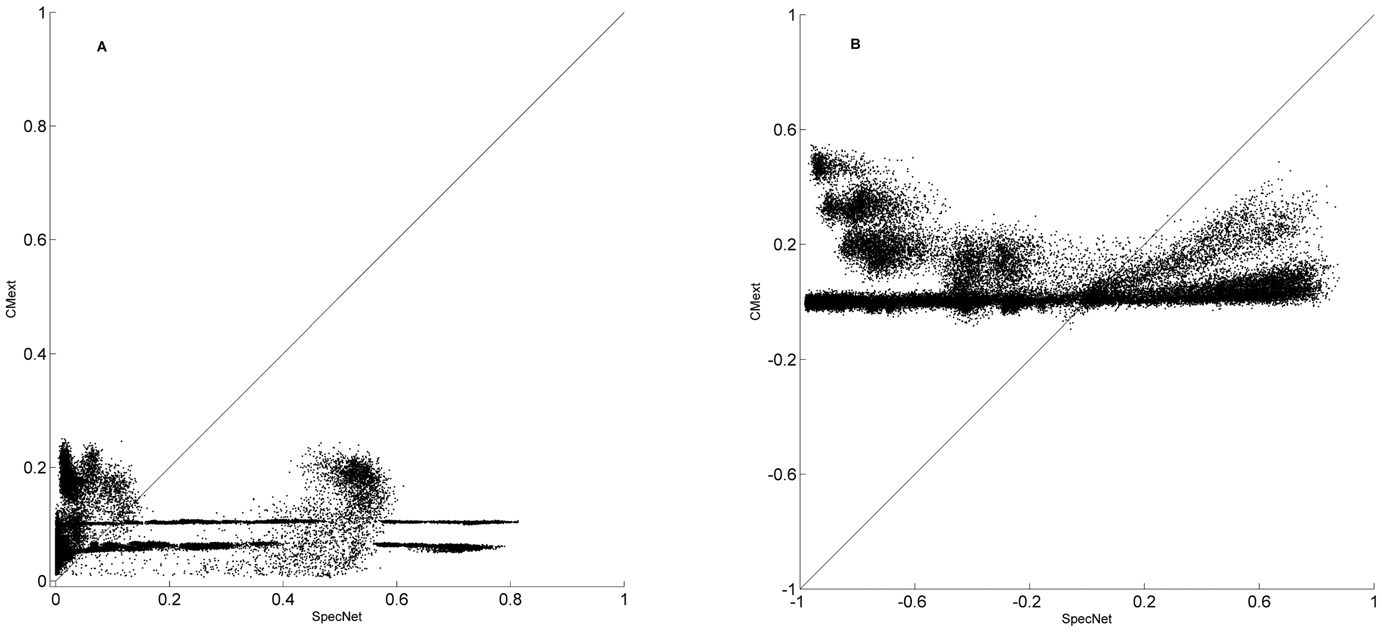
Clustering coefficient and degree assortativity. Clustering coefficient (A) and degree assortativity (B) values generated by the CMext and SpecNet algorithms. The line represents networks where CMext = SpecNet.

SpecNet produced a much larger range of values for all three network measures clustering, degree assortativity, and fragmentation as illustrated in Figure 1. SpecNet shows a superior ability with clustering coefficient ranging from 0 up to 0.81 while CMext generated networks with values from 0 to 0.25. Assortativity values in SpecNet ranged from −0.98 to 0.88 whereas CMext produced networks with degree assortativity up to 0.55 but not lower than −0.09. The fragmentation index for specNet ranged from 0 to 0.96 while a majority of the networks generated by CMext were highly connected and had low fragmentation index values ranging from 0 to 0.20. In Figure 2 we have more specifically compared the ability of the two algorithms to obtain broad ranges of clustering coefficient (Figure 2A) and assortativity (Figure 2B) by plotting CMext values against SpecNet values for each generated network.

**Figure 3:**
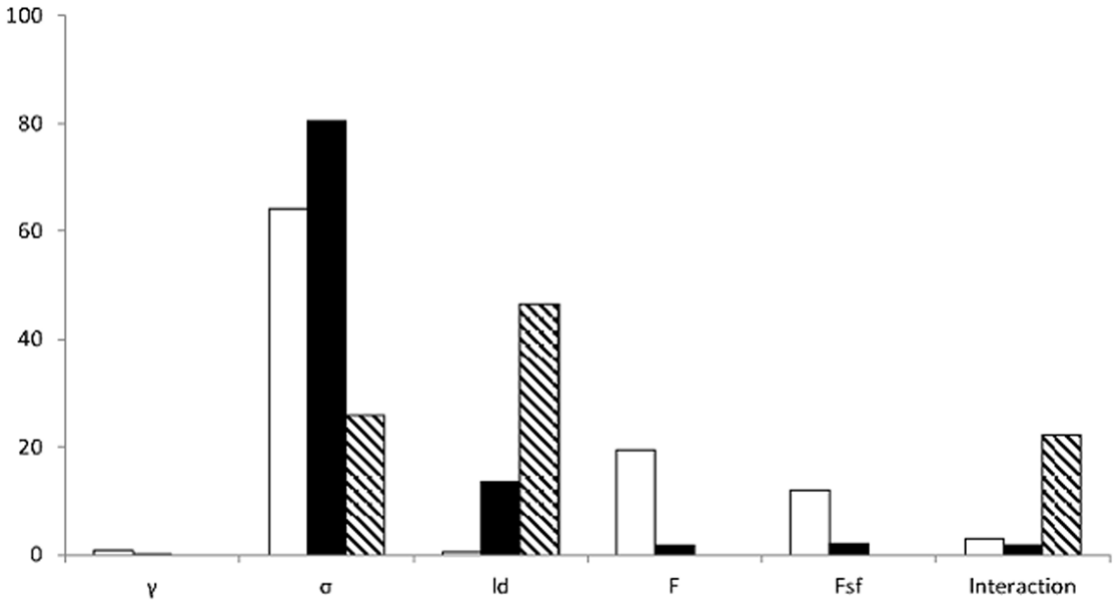
Per cent of mean sum of squares after an ANOVA. Per cent of mean sum of squares after an ANOVA (SpecNet algorithm). White bars for degree assortativity, black bars for average clustering coefficient, and striped bars for fragmentation index.

**Figure 4:**
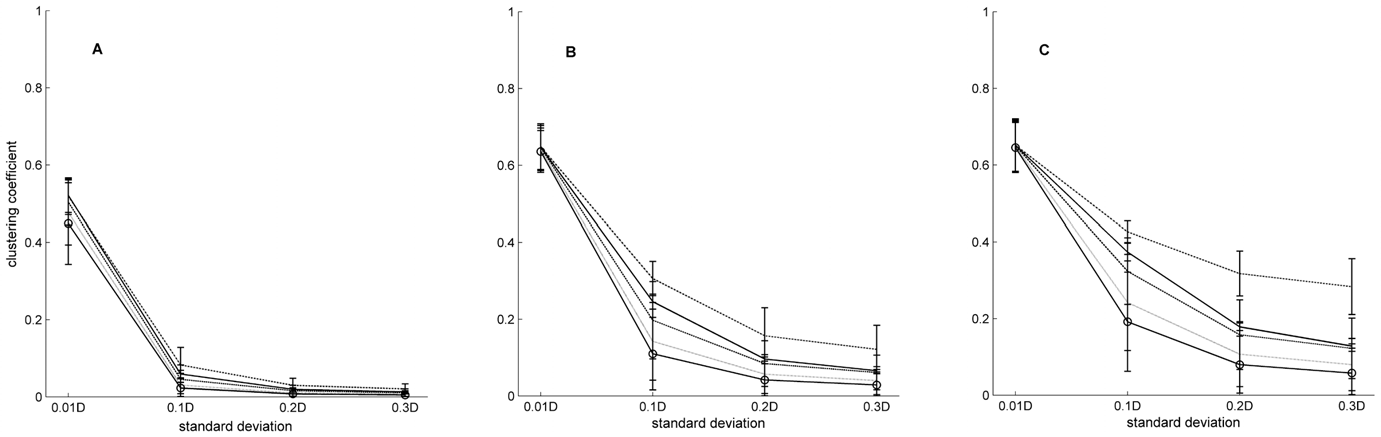
Clustering coefficient depending on standard deviation, link density, and amount of focal nodes. Mean clustering coefficient for different values of *σ* (standard deviation of the dispersal kernel). D = 100√2 is the diagonal of the landscape. Link density (*l_d_*) is 0.01 in (A), 0.05 in (B), and 0.1 in (C). Amount of focal nodes, *F* are: 0 (__), 0.05 (__ _ __), 0.1 (___ _), 0.15 (- - -), and 0.2 (-⊖-). Bars indicate + and − one standard deviation. Each mean was calculated for 750 values made up from combinations of the variables (γ and focal scale factor (*Fsf*)) and replicates of these combinations.

**Figure 5:**
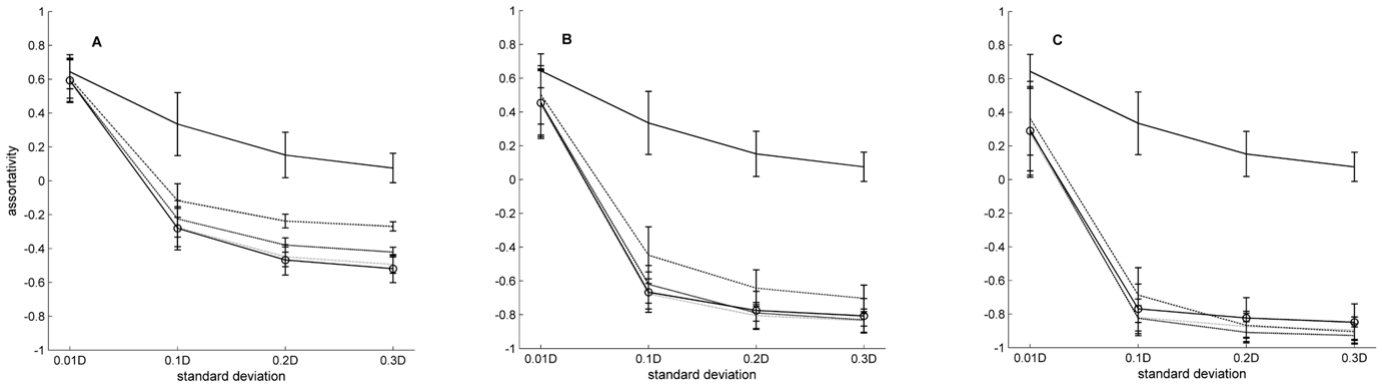
Degree assortativity depending on standard deviation, focal scale factor, and amount of focal nodes. Mean degree assortativity for different values of *σ* (standard deviation of the dispersal kernel). D = 100√2 is the diagonal of the landscape. The focal scale factor (*Fsf*) is 10 in (A), 100 in (B), and 1000 in (C). Each line represents a given amount of focal nodes, *F*: 0 (__), 0.05 (__ _ __), 0.1 (___ _), 0.15 (- - -), and 0.2 (-⊖-). Bars indicate + and − one standard deviation. Each mean was calculated for 750 values representing combinations of the variables (γ and *l_d_*) and replicates of these combinations.

Clustering was best tuned by *σ*, 80.4% of MS, and *l_d_*, 13.6% of MS, assortativity by *σ*, 64.2% of MS, *F*, 19.5% of MS, and *Fsf*, 12.1% of MS, and fragmentation by *l_d_*, 45.6% of MS, and *σ*, 25.9% of MS. Some combinations of parameters also affected the fragmentation, 22.3% of MS, (Figure 3). The clustering coefficient increased with increasing *l_d_* but decreased with increasing *σ* (Figure 4). At a low *l_d_* and a given *σ*, the clustering coefficient was almost the same for different *F* in the networks. The variance in clustering coefficient between networks with different *F* increased with increasing *l_d_* (Figure 4). Assortativity decreased when *σ*, *Fsf*, and *F* were increased (Figure 5).

The continuity parameter, *γ*, in the SpecNet algorithm, had almost no impact at all of any of the three investigated network measures. Nevertheless, when generating networks based on different values of *γ*, we managed to get higher values for all three investigated networks measures, than if using a random node structure. The reason that this was not proved in the ANOVA analysis can be that compared to the total number of networks only a few networks have these high values for the measures. In Table 2 we can also see that the 99% quartile is much higher for the networks including *γ>0*. The fact that parameter, γ, during ANOVA analysis, was shown to have almost no impact of the studied networks measures, is a disparity to the results of our previous study [11] where parameter *γ* appeared to have impact on both assortativity (17% of MS after an anova) and fragmentation index (7% of MS after an anova). We found this disparity a bit odd and necessary to further investigate. Results from a regression tree analysis, in R using mvpart and selecting the best tree with in SE of the overall best using crossvalidiation (xv = “1se”), showed that the parameter *γ* has impact of fragmentation index but no impact on clustering coefficient or assortativity. We also looked at the statistics for three different groups of networks: networks generated by SpecNet with different values of parameter *γ* (Table 1), networks generated by SpecNet with random node structure (*γ* = 0) and networks generated by the algorithm without focal nodes but with different values of *γ* (Table 2). This investigation showed that aggregated node structures (i.e. *γ*>0) are necessary to achieve networks with high values of clustering coefficient (values >0.58), assortativity (values >0.53) as well as fragmented networks (values >0.12) (Table 2).

**Figure 6:**
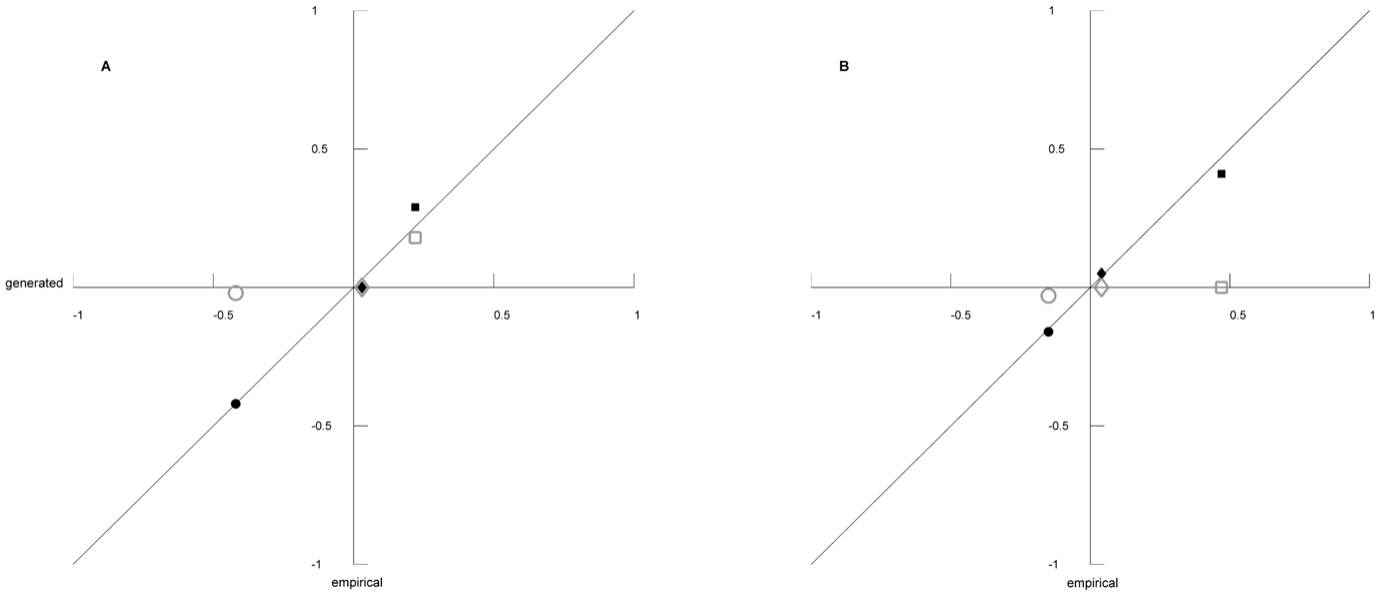
Algorithm performance to regenerate empirical networks. Comparison of the algorithms performances to regenerate two empirical Swedish swine transport networks, the between-farm transport network (A) and the slaughterhouse transport network (B). Values generated by SpecNet are represented as filled markers and values generated by CMext are represented as open markers. Degree assortativity are shown as triangles, clustering coefficient as diamonds, and fragmentation index as squares.

Tables 3 and 4 shows the values for the between-farm transport and the slaughterhouse transport networks respectively that were best reproduced by each algorithm, which for SpecNet represent the parameter combination that had the smallest sum of errors, but for the CMext algorithm represent the replicate with the smallest sum of errors. Mean degree followed the empirical values exactly, since this network measure was given from start from the input data. Table 5 lists the inputs to the SpecNet algorithm that best reproduced the values of the network measures given in Tables 3 and 4. Correlation coefficient of how well algorithms mimicked the transport networks structures to regenerate the desired network structures show high values for SpecNet with 0.9960 for the between-farm transport network and 0.9922 for the slaughterhouse transports network, which indicate good agreement with the empirical values. For CMext the correlation coefficients were lower, 0.7361 for the between-farm transports network and 0.7559 for the slaughterhouse transports network. The SpecNet algorithm thus regenerated the two networks well with respect to all three measures (Figure 6, Tables 3 and 4). The CMext algorithm managed to follow the clustering coefficient and the fragmentation index well for the slaughterhouse transport network (Figure 6A and Table 4). For the between-farm transport network, the algorithm manages to follow the clustering coefficient quite well but it failed to generate a fragmented network (Figure 6B and Table 3). For both empirical networks, the CMext algorithm was highly unsuccessful with regard to degree assortativity (Figure 6, Tables 3 and 4).

**Table 4:** Measures of the network of slaughterhouse transports of swine in Sweden 2008.

		Clustering coefficient	Degree assortativity	Fragmentation index
Empirical network		0.03	−0.42	0.22
SpecNet	Generated	0.00	−0.42	0.29
	Min	0.00	−0.42	0.28
	Max	0.00	−0.33	0.34
	Std	0.00	0.02	0.01
CMext	Generated	0.00	−0.02	0.18
	Min	0.00	−0.02	0.17
	Max	0.00	0.08	0.22
	Std	0.00	0.02	0.01

Empirical values as well as values generated by the SpecNet and CMext algorithms are included. The statistics on generated values from the algorithms are calculated on average values of 200 replicates of each tested combination of parameters for respectively algorithm. Std = standard deviation.

**Table 5:** Input parameters for the SpecNet algorithm for regeneration of empirical networks.

Transport network	*N*	*l_d_*	*γ*	*σ*	*Fsf*	*F*	κ
Between farms	2539	0.0025	1.3247	0.01D	1000	0.2	10/3
Slaughter-house	2539	0.0009	1.3247	0.3D	15	0.2	10/3

For both the between-farm and the slaughterhouse swine transport network, *L_size_* = 175×175. D = 100√2 is the diagonal of the landscape.

## Discussion

Keeling and Eames [39] argued that spatial networks are among the most flexible in generation of different structures. This observation agrees with our results that show that SpecNet can generate networks along nearly the whole range of the theoretical scale of the investigated measures: clustering coefficient, degree assortativity, fragmentation index, and mean degree. However, it is important to note that not all combinations of structures are even theoretical possible, because the network measures are dependent on each other. For example, it is difficult to tune clustering coefficients and degree assortativity separately, since these structures are usually correlated [12]. Furthermore, a network with a high mean degree cannot be as fragmented as a network with a low mean degree.

We choose to generate the node landscape with a more complex method than simply randomly distribute the nodes in the landscape since we found that the node distribution affect the network measures [11]. In networks where the probabilities for links are expected to depend on distances between nodes it is especially important to be able to generate different node distributions. With this method the shape of the node landscape could easily be tuned to more or less aggregated structure just by changing the gamma parameter. The parameter gamma (*γ*) influences the distribution of links between nodes such that an aggregated node landscape (*γ>0*) will result in a skewer link degree distribution than if nodes were randomly placed (*γ = 0*), and that will in turn affect the values of the network measures. It is also possible to estimate values for gamma from real structures, for example transport networks (see [40]). We managed to get higher values for all three investigated networks measures when generating networks based on different values of *γ*, than if using a random node structure. From this we can conclude that our method for generating node structures is necessary to be able to generate high values of clustering coefficient, degree assortativity and/or fragmentation index. Contrary, in the case of generating networks with negative assortativity, it is not necessary to use the complex method with different level of aggregation in the node structure (different values of *γ*) since networks with random node structure (i.e. *γ* = 0) manage to achieve the same low assortativity values. It seems to us that we, in this case, have replaced the need of parameter *γ*, with focal nodes. So, if the aim is to generate networks with negative assortativity, a simpler method, for example random placement of nodes in the landscape, is sufficient. But if the goal is to generate networks with high positive assortativity, high clustering or very fragmented networks, then we recommend using the more complex method which makes it possible to also use aggregated node structures.

The networks generated with the algorithms can contain isolated nodes (i.e., nodes with no links), this is a modification from earlier studies [28], [29], [30] using the CMext algorithm, which did not include networks with isolated nodes. We also choose to let SpecNet create structures that were as different as possible and then used the degree distributions, which were calculated from the output connection matrix for these networks, as input to the CMext algorithm. We did this because the CMext algorithm is primarily developed to reconstruct networks. Still, we could not be certain that CMext covered the whole spectrum of the capability of this algorithm. Since the CMext algorithm, after a set number of triangle building trials, distribute links randomly between nodes, it is not certain either that the networks created by CMext always assess the correct input degree distribution and degree clustering for every replicate. One verification of that the algorithm has a larger capacity than shown in this study is that Weber and Porto (see Figures 4 and 5 in [30]) have shown that the CM algorithm manage to generate networks with assortativity down to about −0.2. It is however important to notice that clustering coefficient and degree assortativity are global measures for the whole network and differ from the degree dependent clustering and assortativity that CMext is adapted to replicate. This may to some extent explain the narrow ranges of the structures generated by CMext. However, it is difficult to define how similar two networks are, in our case we only use a few network measures. For example, it is difficult to determine how different a network with clustering coefficient of 0.04 is compared to a network with clustering of 0.06. One possible way to measure the level of similarity could be to perform disease transmission simulations in such networks and see if and how the outcome coincide between the networks. We will investigate this in another study.

**Figure 7:**
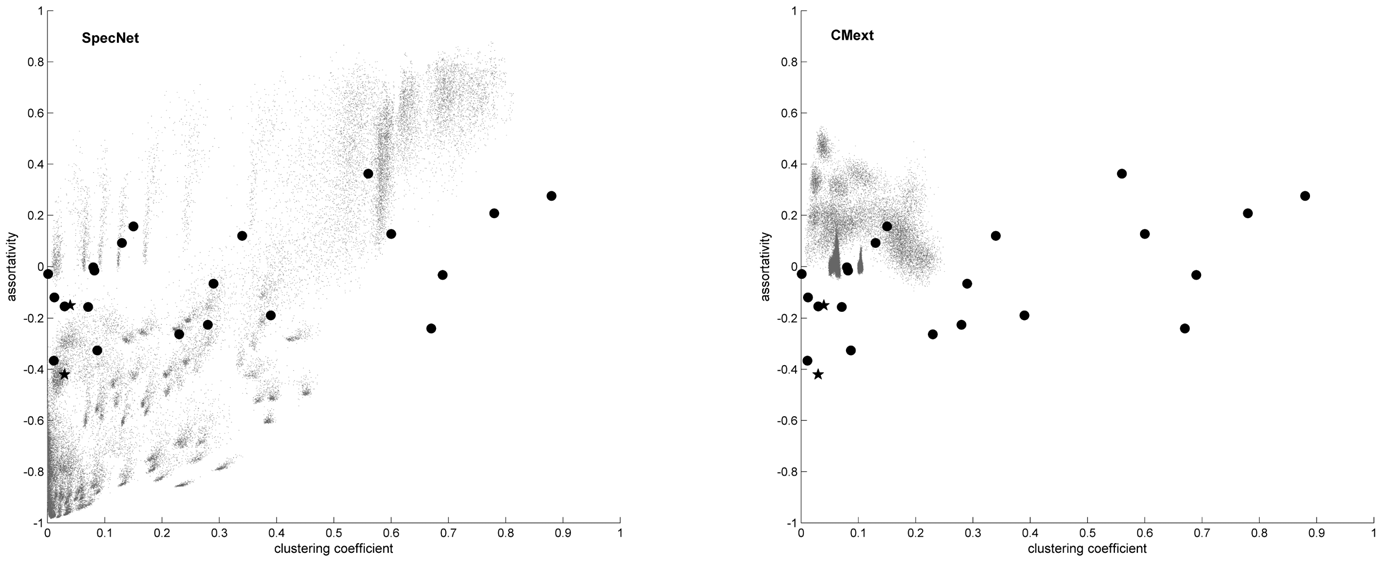
Illustration of clustering and assortativity of real-world networks compared to algorithm generated values. Comparison of clustering and degree assortativity values for real-world networks (filled triangles in both subfigures) and SpecNet generated networks (grey dots) (A) and CMext generated networks (grey dots) (B). Filled stars correspond to the two investigated Swedish swine transport networks, in both subfigures.

To illustrate if values of clustering coefficients and degree assortativity of real-world networks lay in the structure space that SpecNet and CMext are able to generate, such values are compared to corresponding generated values (Figure 7). The real-world networks constituted of the two investigated Swedish swine transport networks, together with additional 21 networks reviewed by Newman [41]. This comparison showed that most of the included real-world networks lie in the structure space which SpecNet is able to generate. But for a few of the real-world networks, the values were located outside the range of SpecNet. These networks were characterized by high clustering in combination with intermediate values of degree assortativty. Only a few of the real-world networks lie in the structure space that CMext has generated in this study.

We have shown that SpecNet generates an exceptionally wide range of network structures. In comparison with an extended configuration model algorithm, CMext, SpecNet was better suited to regenerate the structures of two real animal transport networks used as examples in this study. In addition, to our knowledge there exists no other algorithm that can generate networks with degree assortativity as low as that achieved by SpecNet. SpecNet uses five scalar parameters as input, and the advantage with the algorithm is that these parameters easily can be tuned, one by one, to achieve a desired structure. A further development of SpecNet could be to increase the ability to achieve higher values of clustering coefficient (that is above 0.81). This might be done by using different dispersal kernels for regular and focal nodes.

## References

[1] Asano T (1995) An O(n log log n) time algorithm for constructing a graph of maximum connectivity with prescribed degrees. J Comput System Sci 51: 503–510.

[2] Watts DJ, Strogatz SH (1998) Collective dynamics of ‘small-world’ networks. Nature 393: 440–442. 9623998 10.1038/30918

[3] Barabási A-L, Albert R (1999) Emergence of Scaling in Random Networks. Science 286: 509–512. 10521342 10.1126/science.286.5439.509

[4] Eubank S, Guclu H, Marathe MV, Srinivasan A, Toroczkai Z, et al (2004) Modelling disease outbreaks in realistic urban social networks. Nature 429: 180–184. 15141212 10.1038/nature02541

[5] Keeling MJ (2005) The implication of network structure for epidemic dynamics. Theor Popul Biol 67: 1–8. 15649519 10.1016/j.tpb.2004.08.002

[6] Christley RM, Pinchbeck GL, Bowers RG, Clancy D, French NP, et al (2005) Infection in Social Networks: Using Network Analysis to Identify High-Risk Individuals. Am J Epidemiol 162: 1024–1031. 16177140 10.1093/aje/kwi308

[7] Shirley MDF, Rushton SP (2005) The impacts of network topology on disease spread. Ecol Complex 2: 287–299.

[8] Bansal S, Grenfell BT, Meyers LA (2007) When individual behavior matters: homogeneous and network models in epidemiology. J R Soc Interface 4: 879–891. 17640863 10.1098/rsif.2007.1100 PMC2394553

[9] Boily MC, Asghar Z, Garske Z, Ghani AC, Poulin R (2007) Influence of Selected Formation Rules for Finite Population Networks with Fixed Macrostructures: Implications for Individual-Based Model of Infectious Diseases. Math Popul Stud 14: 237–267.

[10] Badham J, Stocker R (2010) The impact of network clustering and assortativity on epidemic behaviour. Theoretical Population Biology 77: 71–75. 19948179 10.1016/j.tpb.2009.11.003

[11] Håkansson N, Jonsson A, Lennartsson J, Lindström T, Wennergren U (2010) Generating structure specific networks. ACS 13: 239–250.

[12] Badham J, Abbass H, Stocker R (2008) Parameterisation of Keeling's network generation algorithm. Theoret Popul Biol 74: 161–166. 18619479 10.1016/j.tpb.2008.06.002

[13] Keeling MJ (1999) The effects of local spatial structure on epidemiological invasions. Proc R Soc Lond B 266: 859–869. 10.1098/rspb.1999.0716 PMC1689913 10343409

[14] Faloutsos M, Faloutsos P, Faloutsos C (1999) On power-law relationships of the Internet topology. Comp Comm Rev 29: 251–262.

[15] Yook S-H, Jeong H, Barabási A-L (2002) Modeling the internet's large-scale topology. Proc Natl Acad Sci (USA) 99: 13382–13386. 12368484 10.1073/pnas.172501399 PMC129681

[16] González MC, Hidalgo CA, Barabási A-L (2009) Understanding individual human mobility patterns. Nature 453: 779–782. 10.1038/nature06958 18528393

[17] Sevtsuk A, Ratti C (2010) Does urban mobility have a daily routine Learning from the aggregated data of mobile networks Journal of Urban Technology 17: 41–60.

[18] Bell DC, Atkinson JS, Carlson JW (1999) Centrality measures for disease transmission networks. Social networks 21: 1–21.

[19] Badham J, Stocker R (2010) A spatial approach to network generation for three properties: degree distribution, clustering coefficient and degree assortativity. Journal of Artificial Societies and Social Simulation 13: 11.

[20] Newman MEJ, Park J (2003) Why social networks are different from other types of networks. Phys Rev E 68. 10.1103/PhysRevE.68.036122 14524847

[21] Wasserman S, Faust K (1994) Social Network Analysis: Methods and Applications. Cambridge: Cambridge University Press.

[22] Kaiser M, Hilgetag CC (2006) Nonoptimal component placement, but short processing paths, due to long-distance projections in neural networks. PLoS Computational Biology 2: 0805–0815. 10.1371/journal.pcbi.0020095 PMC1513269 16848638

[23] Gastner MT, Newman MEJ (2006) The spatial structure of networks. EPJ B 49: 247–252.

[24] Bender EA, Canfield ER (1978) The asymptotic number of labeled graphs with given degree sequences. JCTA 24: 296–307.

[25] Catanzaro M, Boguñá M, Pastor-Satorras R (2005) Generation of uncorrelated random scale-free networks. Phys Rev E 71. 10.1103/PhysRevE.71.027103 15783457

[26] Molloy M, Reed B (1995) A critical point for random graphs with a given degree sequence. RANDOM STRUCT ALGOR 6: 161–179.

[27] Molloy M, Reed B (1998) The size of the giant component of a random graph with a given degree sequence. CPC 7: 295–305.

[28] Pusch A, Weber S, Porto M (2008) Generating random networks with given degree-degree correlations and degree-dependent clustering. Phys Rev E 77. 10.1103/PhysRevE.77.017101 18351963

[29] Serrano MA, Boguñá M (2005) Tuning clustering in random networks with arbitrary degree distributions Phys Rev E 72. 10.1103/PhysRevE.72.036133 16241541

[30] Weber S, Porto M (2007) Generation of arbitrarily two-point-correlated random networks. Phys Rev E 76. 10.1103/PhysRevE.76.046111 17995064

[31] Christley RM, Robinson SE, Lysons R, French NP (2005) Network analysis of cattle movement in Great Britain. In: Mellor DJ, Russel AM, Wood JLN, editors. Nairn, Scotland 234–244.

[32] Ames GM, George DB, Hampson CP, Kanarek AR, McBee CD, et al (2011) Using network properties to predict disease dynamics on human contact networks. Proc R Soc B 10.1098/rspb.2011.0290 PMC3189367 21525056

[33] Moreno Y, Pastor-Satorras R, Vespignani A (2002) Epidemic outbreaks in complex heterogeneous networks. The European Physical Journal B - Condensed Matter and Complex Systems 26: 521–529.

[34] Barthélemy M (2011) Spatial Networks. Physics Reports 499: 1–101.

[35] Newman MEJ (2010) Networks: An introduction. Oxford University Press.

[36] Newman MEJ (2002) Assortative mixing in networks. Phys Rev Lett 89. 10.1103/PhysRevLett.89.208701 12443515

[37] Borgatti S (2003) The Key Player Problem in Dynamic Social Network Modeling and Analysis: Workshop Summery and papers;. Breiger KC R, Pattison P, editors. National Academy of Sciences Press.

[38] Webb CR (2005) Farm animal networks: unraveling the contact structure of the British sheep population. Prev Vet Med 68: 3–17. 15795012 10.1016/j.prevetmed.2005.01.003

[39] Keeling MJ, Eames KTD (2005) Networks and epidemic models. J Roy Soc Interface 2: 295–307. 16849187 10.1098/rsif.2005.0051 PMC1578276

[40] Mugglestone MA, Renshaw E (2001) Spectral tests of randomness for spatial point patterns. Environmental and Ecological Statistics 8: 237–251.

[41] Newman MEJ (2003) The structure and function of complex networks. SIAM Review 45: 167–256.

